# Pathobiochemical Changes in Diabetic Skeletal Muscle as Revealed by Mass-Spectrometry-Based Proteomics

**DOI:** 10.1155/2012/893876

**Published:** 2012-02-29

**Authors:** Kay Ohlendieck

**Affiliations:** Muscle Biology Laboratory, Department of Biology, National University of Ireland, Maynooth, Kildare, Ireland

## Abstract

Insulin resistance in skeletal muscle tissues and diabetes-related muscle weakness are serious pathophysiological problems of increasing medical importance. In order to determine global changes in the protein complement of contractile tissues due to diabetes mellitus, mass-spectrometry-based proteomics has been applied to the investigation of diabetic muscle. This review summarizes the findings from recent proteomic surveys of muscle preparations from patients and established animal models of type 2 diabetes. The potential impact of novel biomarkers of diabetes, such as metabolic enzymes and molecular chaperones, is critically examined. Disease-specific signature molecules may be useful for increasing our understanding of the molecular and cellular mechanisms of insulin resistance and possibly identify new therapeutic options that counteract diabetic abnormalities in peripheral organ systems. Importantly, the biomedical establishment of biomarkers promises to accelerate the development of improved diagnostic procedures for characterizing individual stages of diabetic disease progression, including the early detection of prediabetic complications.

## 1. Introduction

The incidence of diabetes mellitus has reached epidemic proportions and is one of the great biomedical challenges of the 21st century [1]. Diabetes is classified as a group of chronic metabolic disorders that are characterized by elevated blood glucose levels due to the insufficient production of insulin and/or peripheral insulin resistance [2]. The strict division into type 1 and type 2 diabetes based on an autoimmune etiology versus a primarily metabolic pathology, respectively, has been challenged over the last few years [3] and it has recently been suggested that a continuous spectrum of diabetic disorders exist [4]. Classical type 2 diabetes is a heterogeneous cluster of disorders, whereby both lifestyle and genetic factors play a role in its pathogenesis [2]. An abnormal expression pattern in a number of genes and various proteins has been described to occur in type 2 diabetes [5–7]. Besides impaired pancreatic functioning, peripheral insulin resistance in adipose tissue, the liver, and muscle tissues are the pathophysiological hallmarks of this type of diabetes [8]. Serious medical problems manifest themselves such as diabetic retinopathy [9], diabetic uropathy [10], diabetic foot infection [11], and diabetic nephropathy [12]. Glucotoxic and lipotoxic side effects due to chronic hyperglycemia and hyperlipidemia, respectively, may result in abnormal cellular functions [8]. The impaired uptake of glucose and deleterious accumulation of lipids triggers the principle feature of type 2 diabetes, that is, the progressive dysregulation of carbohydrate homeostasis [13].

With respect to muscle tissues, diabetes affects primarily cardiac cells [14] and the classification of diabetic cardiomyopathy as a distinct clinical entity is currently under intense discussion [15–17]. Heart failure in diabetic patients, independent of coronary artery disease, appears to represent a discrete primary etiology [18]. However, the skeletal musculature is also significantly involved in diabetic complications, that is, contractile weakness, fibre-type changes, decreased oxidative activity and peripheral insulin resistance [19]. Muscle is the most important insulin-dependent glucose sink in the body [20], therefore, impaired hormonal signaling has a deleterious effect on glucose uptake. The detailed biochemical characterization and proteomic establishment of distinct shifts in metabolic and signaling pathways in diabetic fibres might provide mechanistic insights into the underlying causes of (i) abnormal glucose metabolism [21] and peripheral insulin resistance [22, 23], (ii) diabetes-associated loss of skeletal muscle mass and contractile strength [24, 25], which is especially prevalent in aged patients [26, 27], (iii) muscle mitochondrial dysfunction and the role of mitochondrial deficits in overall disease progression [28–30], and (iv) slow-to-fast muscle transitions and reduced oxidative enzyme activity in muscle of type 2 diabetic subjects [31].

The fact that drastic lifestyle modifications, such as an altered diet and increased physical activity [32], have a profound influence on insulin resistance and the onset of diabetic complications [33, 34] clearly demonstrates the crucial role of skeletal muscle metabolism in diabetes mellitus. Since a single bout of exercise triggers a substantial increase in whole-body glucose disposal, mediated by the translocation of the crucial glucose transporter GLUT4 to the surface membrane system in contracting skeletal muscles [35], exercise training should play a key role in the prevention and treatment of diabetes [36–38]. To better understand these beneficial effects of physical activity, it will be essential to increase our molecular understanding of how exercise utilizes different signaling mechanisms as compared to hormonal regulation of glucose uptake. Genomic, proteomic and metabolomic approaches are highly suited to the conduction of such investigations and might determine the best way to counteract diabetic complications in the skeletal musculature.

## 2. Mass-Spectrometry-Based Skeletal Muscle Proteomics

Modern proteomics attempts to identify, catalogue, and characterize the entire protein constellation of specific cell types, tissues, or biological fluids using large-scale separation methods, such as two-dimensional gel electrophoresis or liquid chromatography, and high-throughput identification approaches focusing mostly on mass spectrometric techniques [39]. Proteomic studies of plasma and cells with mostly soluble components have revealed excellent coverage of protein complements. However, the dynamic range of protein expression in complex tissues and the large number of hydrophobic protein species that are present in membranous systems are major challenges for routine proteomic surveys [40]. Immuno-depletion of abundant components, equalization methods, or subcellular prefractionation can be used to overcome some of the problems associated with wide concentration ranges of proteins by reducing sample complexity [41–43]. The analysis of the membrane proteome can be achieved by employing detergent phase extraction methods [44], on-membrane digestion protocols [45], or filter-aided sample preparation [46] in order to include hydrophobic elements in proteomic surveys.

However, aside from these technical problems, several physiological, cell biological, and biochemical parameters make the proteomic analysis of skeletal muscle an especially difficult task. In order to cover as many proteins as possible in proteomic studies, the initial extraction process of tissue samples is crucial. Since muscle fibres are rigid structures that are embedded in a complex system of connective tissue, these biological properties make it an extremely tough tissue to homogenize. Approximately half of the muscle protein content consists of the contractile apparatus with its various isoforms of myosin light chains, myosin heavy chains, troponins, tropomyosins, and associated proteins. Due to the high density of these proteins, a certain degree of cross-contamination of protein spots cannot be avoided during two-dimensional gel electrophoresis. In addition, a large number of very high-molecular-mass proteins exist in skeletal muscle fibres, with some of them being susceptible to proteolytic degradation [47]. The occurrence of protein fragments has therefore to be taken into account in proteomic analyses.

With respect to the under-representation of integral proteins in most proteomic investigations of crude tissue extracts, it is important to stress that skeletal muscles exhibit a highly elaborate membrane system, consisting of the sarcolemma, transverse tubules, triad junctions, the sarcoplasmic reticulum, and abundant organelles such as mitochondria. Since mature skeletal muscle tissues are heterogeneous in composition, it has to be taken into account that total extracts contain muscle fibre proteins and components derived from nerves, capillaries, basal lamina, and satellite cells [48]. The musculature is physiologically a highly adaptable system with the activity of the critical nerve-muscle connection at the neuromuscular junction dictating gene expression levels based on dynamic changes in neuromuscular activity. Thus, ideally proteomic studies have to take the protein complement of motor neuron populations into account for comprehensive investigations of the muscle proteome [49]. Predominantly fast-twitching and slow-twitching fibres, as well as its many subtypes and hybrid forms, differ considerably in their proteomic constellation and adaptive capacity [50–52]. An enormous variety of myosin chain combinations can be found in developing, maturing, and aging skeletal muscles [53]. In this respect, mass-spectrometry-based proteomics is an ideal tool to differentiate subtypes of muscle fibres within a crude extract based on the unequivocal identification of myosin light and heavy chain combinations.

**Figure 1:**
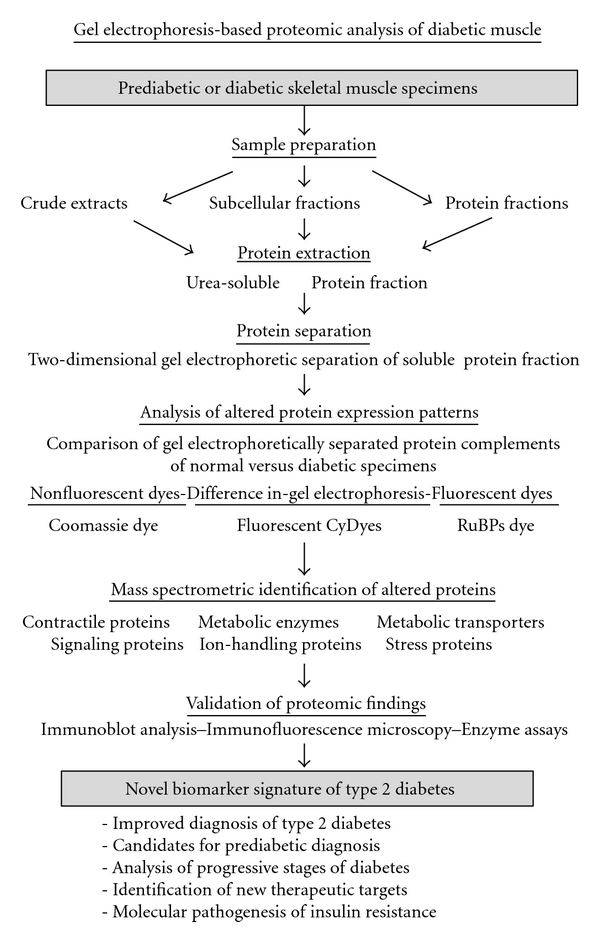
Proteomic profiling strategy to evaluate the effect of type 2 diabetes on skeletal muscle tissues. Shown is a flowchart of the proteomic workflow to identify new protein factors involved in the molecular pathogenesis of insulin resistance, abnormal cellular signaling, and contractile weakness in diabetic skeletal muscle tissues. Listed are the various analytical steps of mass-spectrometry-based proteomic surveys of muscle samples, such as sample preparation, protein extraction, gel electrophoretic separation, densitometric scanning, mass spectrometric identification of novel biomarkers, and the independent validation of proteomic data by immunoblotting, enzyme assays, and immunofluorescence microscopy.

The cellular and functional integrity of muscles depends heavily on constant loading and proper activity levels. On the one hand, chronic electrostimulation or physical training triggers profound changes in the protein expression pattern of muscles [54–57], and on the other hand, disuse leads inevitably to muscular atrophy [58–60]. Proteomics can be employed to catalogue and characterize subtle changes in the abundance and/or isoform expression during muscle alterations. Recent scientific achievements and technical challenges for future proteomic studies have been critically examined in several reviews [47, 48, 61–63]. See Figure 1 for the proteomic workflow of analyzing diabetic muscle tissues using a gel electrophoresis-based approach.

## 3. Proteomic Profiling of Diabetes Mellitus

 Over the last few years, mass-spectrometry-based proteomics has been established as a crucial analytical tool for biomarker discovery in the heterogeneous pathology of diabetes [64–67]. Proteomic studies have focused on diabetic nephropathy [68–70], diabetic peripheral neuropathy [71] and diabetic retinopathy [72, 73], as well as glucotoxicity and abnormal islet function [74–76]. Although skeletal muscle fibres also play a major role in type 2 diabetes, only a few proteomic surveys have addressed the issue of diabetes-related changes in muscle protein expression levels, as listed in Table 1. The sections below critically examine the impact of recently published proteomic studies of type 2 diabetic skeletal muscle tissues [77–85].

### 3.1. Proteomic Profiling of Human Muscle in Type 2 Diabetes

 The occurrence of diabetes and cardiovascular disease has reached epidemic rates and there is a clear association of these disorders with obesity-related complications. Impaired insulin-mediated glucose uptake in skeletal muscle fibres is related to high levels of circulating free fatty acids, an increased intramyocellular lipid content, diminished mitochondrial functioning and an overall weakened metabolic flexibility [21]. Pathobiochemical pathways that mediate chronic low-grade inflammation represent the most likely molecular link between obesity, diabetes, and cardiac disease [86]. To properly study the role of obesity and diabetes in disturbing skeletal muscle metabolism and to evaluate the association between these two pathologies, a comparative proteomic survey would ideally determine global protein expression differences between age- and gender-matched, lean, nondiabetic subjects, and individuals that are classified as obese and diabetic, obese and nondiabetic, and nonobese and diabetic. Since the availability of large numbers of clinical human tissue samples for comparative biochemical surveys is a challenge, so far only a few proteomic studies have addressed this fundamental question using human muscle specimens [77–81].

Two-dimensional gel electrophoresis in combination with MALDI-ToF MS analysis identified a small number of potential muscle-associated biomarkers for type 2 diabetes [77, 78]. The proteomic comparison of muscle samples from 10 diabetic patients versus 10 healthy gender- and age-matched controls revealed 8 potential markers of type 2 diabetes in the fasting state. This included the *β*-subunit of ATP synthase, isoforms of myosin light chain MLC2, creatine kinase, the *α*1(IV)-chain of collagen, glucose-regulated protein GRP78, the molecular chaperone Hsp90, and the glycolytic enzyme phosphoglucomutase [77]. Increased levels of the heat shock proteins Hsp90 and GRP78 indicated increased cellular stress in diabetic muscle tissue. Elevated concentrations of phosphoglucomutase suggested an increased glycolytic-to-oxidative ratio in diabetic skeletal muscle during the fasting state. Altered levels of the enzymatic interconversion of glucose-1-phosphate and glucose-6-phosphate possibly play a role in altered glycogen and glucose metabolism in diabetes [77]. Importantly, the catalytic *β*-subunit of ATP synthase was shown to exhibit abnormal phosphorylation in insulin-resistant muscle [77, 79]. The biochemical comparison of muscle samples from cohorts of healthy lean controls versus obese individuals versus patients afflicted with diabetes showed that the *β*-subunit of ATP synthase is phosphorylated at multiple sites and that abnormal phosphorylation patterns are present in diabetic muscle [79]. The human ATPsyn-*β* molecule exhibits phosphorylation sites at Thr213, Tyr230, Tyr269, Thr312, Tyr361, Tyr395, and Thr475, whereby Thr213 is located in the nucleotide-binding region of this enzyme. In muscle samples from obese and diabetic individuals, Thr213 and Tyr361 in the *β*-subunit of ATP synthase showed increased basal phosphorylation levels. In addition, various enzymes involved in oxidative phosphorylation were decreased in diabetic specimens [79]. Thus, dysregulation of ATP synthesis probably triggers perturbations in mitochondrial functions and may be a contributing factor in type 2 diabetes.

Recently, a more comprehensive proteomic study of diabetic muscle was carried out by one-dimensional gel electrophoresis and HPLC-ESI-MS/MS analysis. Hwang et al. [80] compared the protein constellation of small percutaneous vastus lateralis muscle biopsies from lean, obese and type 2 diabetic subjects. This proteomic approach had previously been successfully applied to catalogue the human skeletal muscle protein complement and lead to the identification of 954 proteins in normal human vastus lateralis [93]. In the diabetic muscle study, of 1,218 proteins assigned by mass spectrometric identification, 400 protein species were present in at least half of the 24 volunteers. A significant increase or decrease was established in 15 muscle proteins [80]. While the expression of mitochondrial elements was shown to be lower in diabetic muscle, several molecular chaperones appear to be higher in insulin-resistant fibres. Increased levels of the molecular chaperones Hsp90, T-complex protein, and chaperonin, as well as the enzyme protein disulfide isomerase, agree with the idea of increased cellular stress in diabetic fibres [80]. An altered concentration of *α*-actinin and its binding-protein myozenin and a specific isoform of myosin indicate changes in skeletal muscle structure. Drastically decreased mitochondrial proteins were identified as ubiquinol-cytochrome C reductase and cytochrome c oxidase, confirming a perturbed mitochondrial functioning in type 2 diabetes [80]. Reduced expression levels of genes encoding mitochondrial enzymes have previously been demonstrated by microarray studies [94]. Interestingly, the proteomic survey of rectus abdominus muscle from obese and morbidly obese women strongly suggests a compensatory glycolytic shift [87]. Increased glycolytic activity might represent a metabolic rescue to counteract mitochondrial dysfunction during progressive obesity- or diabetes-related impairments in oxidative muscle metabolism. The comparative proteomic analysis of mitochondria from insulin-sensitive versus insulin-resistant vastus lateralis muscle suggests an altered density of key enzymes involved in fat oxidation which might be an important contributory aspect of lipid accumulation in diabetes [79].

### 3.2. Proteomic Profiling of Animal Models of Type 2 Diabetes

Since abnormal skeletal muscle functioning is a major feature of type 2 diabetes, it can be expected that distinct changes occur in the muscle proteome during the development of insulin resistance and diabetes. Established animal models with key symptoms of diabetes are, therefore, an ideal experimental tool to study global alterations in the abundance, isoform expression pattern, and/or posttranslational modifications of muscle proteins.

#### 3.2.1. Animal Models of Type 2 Diabetes

It is clearly evident from the large number of publications on animal model research covering diabetes mellitus that studying laboratory animals had a significant impact on understanding diabetic pathogenesis, testing novel drugs, and evaluating toxic side effects [95–99]. Inbred animal strains show genetically considerably less interindividual differences than human subjects. Hence, a lower number of experimental repeats is capable of producing meaningful sets of analytical data. Since diabetes is a disease of both genetics and lifestyle that shows a complex pattern of symptoms affecting various tissue and organ systems [4], no one diabetes-related animal model is sufficient to study all of the clinical aspects of the metabolic symptoms seen in patients. Therefore, a major research drive is to generate new diabetes models focusing on the various aspects of this complex metabolic disorder, such as neuropathy, retinopathy, uropathy, cardiovascular problems, and insulin resistance in skeletal muscle, as well as the overall impact of glucotoxicity on body homeostasis [2]. Enormous progress has been made by studying models of type 1 diabetes, such as streptozotocin-induced diabetes in rats [100], as well as type 2 diabetes including the nonobese Goto-Kakizaki rat [101] and the obese Zucker rat [102].

With the emergence of genomics and proteomics, the search for novel genes and protein factors involved in the pathophysiology of diabetes has been decisively improved. The proteomic profiling of animal disease models is highly suitable for the identification of new biomarker signatures, for the development of superior diagnostics, and for the evaluation of novel treatment options to counteract diabetes-related side effects in skeletal muscle tissues. As reviewed by Resjö et al. [103], studying animal models of diabetes using proteomics has provided unparalleled mechanistic insights into the development of insulin resistance and has revealed new biomedical opportunities for diabetes prevention strategies. However, it is crucial to keep in mind that related animal species may differ markedly with regard to drug effects, toxic side effects, dietary exposures, and diabetes development, strongly suggesting that studying a single animal system is not sufficient to properly predict human responses. Although laboratory animals differ from humans in many physiological and biochemical responses which may affect the degree of cellular pathogenesis, studies of diabetic animals can be helpful to human diabetes predictions and prevention.

As discussed in general by Doran et al. [104], a good animal model of diabetes should (i) closely resemble the etiology of human diabetes in onset, progression, complexity, and severity, (ii) develop all or most of the multifactorial aspects usually observed in advanced stages of human diabetes, (iii) mimic the basic mechanisms of human physiology and metabolism that are important for insulin resistance, (iv) be suitable for genetic manipulations and cell-based treatment strategies, and (vii) be large enough to yield sufficient amounts of biological samples for extended biochemical analyses or to facilitate physiological procedures. The initial findings from comparative animal proteomics can be extremely useful for making an informed decision on the design of large-scale clinical proteomics investigation [104]. In the future, genetically engineered animal models promise to be extremely helpful for determining the pharmacological efficacy of novel diabetic drugs. Thus, animal model research will be an important prerequisite for proper drug selection prior to the establishment of large-scale clinical trials for the treatment of diabetes.

#### 3.2.2. Proteomic Profiling of Skeletal Muscle from Diabetic Animal Models

Since mass-spectrometry-based proteomic studies of potential changes in the skeletal muscle proteome have mostly focused on the Goto-Kakizaki rat [82–84], the suitability of this animal model of type 2 diabetes is briefly described. The general diabetic status of mature Goto-Kakizaki rats is exemplified by the fact that their blood glucose levels are significantly elevated, but that the concentration of nonfasting plasma insulin is not affected [101, 105, 106]. With respect to skeletal muscle tissues of the Goto-Kakizaki rat, numerous studies have clearly shown diabetes-related abnormalities, including (i) an inhibition of insulin receptor autophosphorylation [107], (ii) abnormal functioning of insulin signaling intermediates [108, 109], (iii) an altered subcellular localization and diminished recruitment of the main glucose transporter GLUT4 [110], (iv) cytoskeletal defects in the sarcolemmal dystrophin-dystroglycan complex [111], (v) a significantly reduced percentage of oxidative fibres [112], and (vi) abnormal mitochondrial functioning [113]. Hence, chronically impaired insulin signaling and associated downstream alterations in skeletal muscle tissues make adult Goto-Kakizaki rats a suitable animal model of type 2 diabetes.

The proteomic profiling of crude muscle extracts and subcelluar fractions from the Goto-Kakizaki rat has employed different protein staining methods for the visualization of protein spots in two-dimensional gels, that is, colloidal Coomassie Blue [82], fluorescent RuBPs [82, 83], and fluorescent CyDyes for difference in-gel electrophoretic analysis [83, 84]. The different labeling techniques clearly showed variations in their dynamic visualization range, so proteomic findings from the 3 dyes used for densitometric scanning could be combined for a more complete coverage of changes within the diabetic muscle proteome. Colloidal Coomassie staining, routinely used for reliable protein detection following gel electrophoresis [114, 115], and fluorescence difference in-gel electrophoresis, which represents one of the most powerful biochemical tools for the comparative analysis of protein complements [116–118], resulted in the detection of 929 [82] and 1734 [83] protein spots, respectively. The silver staining technique and protein labeling with the fluorescent RuBPs dye (ruthenium bathophenanthroline disulfonate) [119] were employed as independent visualization methods for the verification of key findings in altered protein expression patterns [82, 83]. The mass spectrometric identification of distinct changes in proteins revealed that the nonobese diabetic phenotype exhibits a generally perturbed protein expression pattern. Affected protein species were shown to be associated with the contractile apparatus, the antioxidant defense system, detoxification mechanisms, the cellular stress response, glucose metabolism, fatty acid utilization, nucleotide metabolism, and amino acid metabolism [82–84].

The gel electrophoresis-based proteomic survey of normal versus nonobese diabetic rats showed an altered expression profile for muscle-associated proteins that are involved in lipolytic catabolism, the citric acid cycle, oxidative phosphorylation, glycolysis, nucleotide metabolism, carbon dioxide removal, oxygen transportation, amino acid catabolism, and cellular detoxification mechanisms [82]. Mass spectrometry identified the enzymes with the highest decrease as carbonic anhydrase CA3 and 3-hydroxy-isobutyrate dehydrogenase. The CA3 isoform of carbonic anhydrase mediates the vital conversion of CO_2_ into carbonic acid in skeletal muscle tissues. Hence, the CO_2_-removal mechanism appears to be impaired in diabetic muscles. The degradation of leucine, valine, and isoleucine involves the activity of hydroxy-isobutyrate, whereby the resulting carbon skeleton is utilized as a metabolic substrate for the generation of energy. The reduction of this rate-limiting enzyme suggests that the degradation pathway of amino acids is diminished in diabetic fibres.

The proteomic analysis of nonobese diabetic muscle revealed a differential effect on glycolytic enzymes. The concentration of enolase appears to be decreased, while aldolase and phosphoglucomutase showed elevated levels in muscle preparations from the Goto-Kakizaki rat [82]. Increased expression of phosphoglucomutase in diabetic rat muscle agrees with the above-discussed findings of an elevated density of this key glycolytic enzyme in human diabetic skeletal muscle [77]. Altered levels of glycolytic enzymes will have a profound effect on the metabolic flux in diabetic muscle. However, numerous glycolytic enzymes were shown to be multifunctional, making it difficult to conclude that peripheral insulin resistance in muscle directly affects the expression of select members of the 10-enzyme system of muscle glycolysis.

Interestingly, increased levels of adenylate kinase isoform AK1 and monoglyceride lipase were shown to exist in diabetic gastrocnemius muscle from the Goto-Kakizaki rat [82]. Changes in adenylate kinase could be interpreted as diabetes-related alterations in nucleotide metabolism and agrees with findings from the proteomic analysis of obese skeletal muscle [87]. The metabolic enzyme monoglyceride lipase catalyses a key step in the hydrolysis of stored triglycerides [120] and its increased abundance in diabetes may represent compensatory energy utilization by the lipolytic pathway in glucose-starved, nonobese muscle fibres. In the absence of alternative signaling mechanisms, such as exercise-induced glucose transport, insulin resistance clearly generates a lack of glucose removal from the circulatory system. Decreased glucose uptake by skeletal muscle tissues then triggers a metabolic knock-on effect on other biochemical pathways such as gluconeogenesis, triacylglycerol hydrolysis, fatty acid oxidation, and ketone body formation [21]. Thus, the dysregulation of metabolism in diabetic muscle fibres is a highly complex pathology and proteomics has provided additional information of hitherto undetected changes in protein factors that may play a contributory role in type 2 diabetes. In the long term, a more complete comprehension of these complex metabolic alterations will improve our understanding of the overall pathobiochemical processes that lead to peripheral insulin resistance and diabetes-related muscle weakness.

In addition to newly detected changes in metabolic enzymes, the fluorescence difference in-gel electrophoretic analysis of diabetic muscle revealed significantly increased levels of the small stress proteins *α*B-crystallin and Hsp27 [83]. The apparent upregulation of molecular chaperones indicates an enhanced cellular stress response in the diabetic phenotype. In general, stress proteins protect muscle cells during unfavorable conditions, such as extreme hyperthermia, ischemic damage, hypoxic insult, exercise-induced fibre damage, traumatic injury, and in disease-associated muscle degeneration [121]. The family of small heat shock proteins is characterized by a conserved carboxy-terminal sequence, the alpha-crystallin domain [122]. Cytoprotective proteins containing this 90-residue domain are specifically induced during injury to contractile fibres [123]. Muscle-specific molecular chaperones counteract deleterious protein aggregation and specifically modulate intermediate filament assembly [124]. The elements of the diabetes-associated increase in the cellular stress response might be useful candidates for the establishment of a comprehensive biomarker signature of type 2 diabetes.

Since mitochondrial dysfunction in skeletal muscle has been implicated in the progression of diabetic pathology [28–30], it is of considerable interest to determine how the mitochondrial proteome is altered in diabetic fibres. Mitochondria play a key role in cell cycle progression, calcium signaling, intermediary metabolism, protein transport, regulation of apoptosis, and energy generation via oxidative phosphorylation [125]. Altered protein expression levels or functional modifications of mitochondrial proteins are intrinsically involved in numerous development processes, the natural decline in body systems during aging, and the progression of many different pathologies [126]. As reviewed by Distler et al. [127], the mitochondrial proteome contains approximately 1,500 individual protein species. This relatively manageable number of proteins can be routinely analysed by proteomic technologies, establishing subcellular proteomics as a suitable analytical method to evaluate global alterations in the mitochondrial protein complement. The fluorescence difference in-gel electrophoretic analysis of isolated mitochondria from diabetic gastrocnemius muscle showed changed concentration levels of a variety of metabolic enzymes [84]. An altered expression of NADH dehydrogenase, cytochrome b-c1 complex, isocitrate dehydrogenase, pyruvate dehydrogenase, and ATP synthase might trigger a diabetes-dependent impairment of mitochondrial oxidative phosphorylation in Goto-Kakizaki rat muscle. Thus, mitochondrial abnormalities appear to play a contributory role in the molecular pathogenesis of type 2 diabetes and may be associated with the progressive development of insulin resistance [128]. With respect to human diabetes, the recent successful phosphoproteomic cataloguing of human muscle mitochondria by Zhao et al. [129] has demonstrated that it will be possible to study functional mitochondria from human muscle by proteomics in the future. Such mass-spectrometry-based studies promise to determine the potential role of altered mitochondrial protein expression levels and/or posttranslational modifications in human type 2 diabetes.

Besides proteomic surveys of skeletal muscles from nonobese type 2 diabetic animal models, mass spectrometric methodology was also applied to the evaluation of fenofibrate-fed type 2 diabetic OLETF rats [85], type 1 diabetic BB-DP rats [130], streptozotocin-induced type 1 diabetic mice [88], and animal models of obesity with a potential for prediabetic complications, such as C57BL/6 mice on a high-fat diet [89], interleukin receptor-knockout mice on a high-fat diet [90], capsaicin-treated obese rats [91], and obesity-prone OP rats on a high-fat diet [92]. The pharmacological substance fenofibrate is an agonist of the peroxisome proliferator-activated class of receptors, which are involved in the regulation of carbohydrate and lipid metabolism. A functionally unknown muscle protein named C11orf59 was shown to be markedly increased in a fenofibrate-dependent manner in diabetic OLETF rats [85]. Obesity appears to have a profound influence on the skeletal muscle proteome, triggering a disturbed expression pattern in various metabolic enzymes [89–91]. Thus, the loss of metabolic homeostasis probably underlies obesity-associated disorders, such as the prediabetic syndrome and type 2 diabetes.

**Figure 2:**
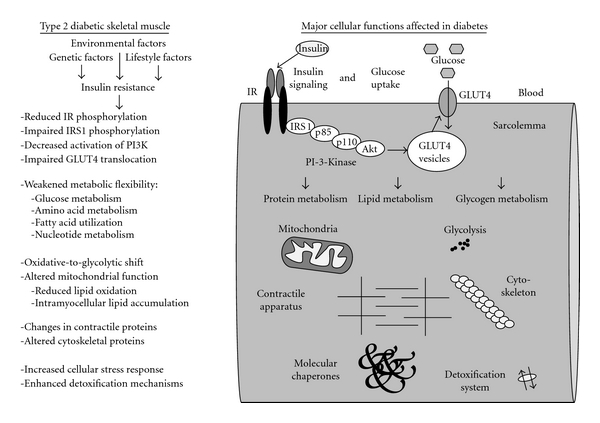
Overview of changes in diabetic skeletal muscle as revealed by mass-spectrometry-based proteomics. Shown is a diagram of a skeletal muscle fibre outlining sarcolemmal proteins involved in insulin signaling and glucose uptake, as well as major cellular mechanisms affected in diabetic muscle tissues. Listed are established diabetes-related impairments of insulin receptor (IR) phosphorylation and abnormal signaling and transporter recruitment involving IRS1, p85, p110, Akt, PI-3-kinase, and glucose transporter isoform GLUT4. Proteomic profiling of muscle tissues from patients and established animal models of type 2 diabetes have revealed changes in components downstream from these plasmalemmal signaling cascades, including proteins involved in mitochondrial metabolism, glycolysis, contractile apparatus, detoxification mechanisms, cellular stress response, glucose metabolism, fatty acid utilization, nucleotide metabolism, and amino acid metabolism.

The main findings of proteomic surveys of diabetic muscle preparations are summarized in Figure 2. Although only a limited number of studies have been published that used high-resolution two-dimensional gel electrophoresis or liquid chromatography in combination with mass spectrometry for the analysis of type 2 diabetes, a large number of changed muscle proteins involved in various cellular functions have already been identified. The future application of detailed biochemical, physiological, and cell biological analyses will be crucial to determine the general suitability of these new potential biomarkers as reliable indicators of downstream effects of insulin resistance and disease progression. This will not only be important for improving our biomedical understanding of the molecular pathogenesis that leads to diabetes mellitus but also be helpful for the improved design of diagnostics and the identification of novel therapeutic targets. As recently outlined by Herder et al. [131], the establishment of novel biomarkers for the prediction of type 2 diabetes is crucial to the identification of high-risk individuals who could benefit from targeted preventive measures. In this respect the, albeit limited, application of mass-spectrometry-based proteomics has shown that this highly specialized biochemical technology can make a valuable contribution to the general field of diabetes research.

## 4. Conclusion

Cardiovascular diseases, obesity-related disorders affecting multiple organ systems, the metabolic syndrome, and diabetes mellitus affect hundreds of millions of patients worldwide. These epidemiological facts warrant detailed biomedical studies into the molecular and cellular mechanisms of these crippling diseases. It is hoped that a better understanding of the molecular pathogenesis of common human disorders will help in the identification of novel therapeutic targets. In the long term, this should translate into the development of superior pharmacological approaches and better clinical treatment options. The etiology of type 2 diabetes appears to be influenced by both genetic and environmental factors, making it a great challenge to determine the causative interplay of genetic susceptibilities, pathophysiological aspects, and external features. A change in lifestyle, such as increased physical activity and improved nutritional regimes, can reverse some of the diabetic symptoms such as insulin resistance. Diabetes is clearly a heterogeneous group of disorders and can cause extremely serious side effects, such as cardiomyopathy, stroke, lower limb amputation, kidney failure, blindness, and skeletal muscular weakness. In the older population, type 2 diabetes can result in significantly decreased muscle strength and may thus contribute to the development of physical disability in the elderly. Sarcopenia of old age, the natural age-related decline in skeletal muscle mass and strength, can be exacerbated by the negative influence of the diabetic phenotype on muscle metabolism. It is, therefore, of the uttermost urgency to devise novel approaches to counteract diabetes-related muscle weakness and abnormal hormone signaling in of the most crucial insulin-dependent organs for glucose disposal. Over the last few years, mass-spectrometry-based proteomics has been applied to diabetes research as an unbiased analytical tool for the global determination of abnormal protein expression patterns in diabetic muscle tissues. The proteomic profiling of both biopsy samples from diabetic subjects and muscle extracts from established animal models has resulted in the identification of a variety of new potential markers of diabetes. The biomarker signature of muscle-related changes due to diabetes includes components that are associated with the actomyosin apparatus, the cellular stress response, glucose utilization, fatty acid oxidation, and other metabolic pathways. Interestingly, a very recent proteomic study of human diabetic muscle clearly supports the idea of oxidative-to-glycolytic shifts in energy metabolism of the diabetic phenotype [132]. In addition, Thingholm et al. [133] have recently examined human myotubes from type 2 diabetic subjects and identified adenosine deaminase as a contributing factor in diabetes. The identification and characterization of disease stage-specific indicators is especially crucial for the evaluation of the prediabetic phase. Insulin resistance in skeletal muscle represents one of the main features of diabetes-related dysregulation and probably develops during a relatively early phase of the prediabetic state. Although low levels of insulin resistance can probably be partially compensated by increased hormonal secretion via enhanced pancreatic beta-cell activity in early type 2 diabetes, at more advanced stages of the disease, beta-cell failure occurs resulting in an insufficient concentration of circulating insulin to prevail over signaling defects in muscle tissue sensitivity. Building on the findings of the initial proteomic surveys conducted, as outlined in this review, future mass-spectrometry-based investigations promise to identify new protein factors that will be indispensable for the improvement of diagnostic techniques that could monitor diabetic progression, and the discovery of superior therapeutic targets to eliminate peripheral insulin resistance and diabetes-associated contractile weakness.

## Figures and Tables

**Table 1:** List of major proteomic profiling studies of skeletal muscle tissues from prediabetic and diabetic patients or animal models of type 2 diabetes.

Proteomic study	New potential biomarkers	References
Proteomic analysis of human vastus lateralis muscle from type 2 diabetic subjects	Select number of potential markers of diabetes; abnormal phosphorylation of ATP synthase; elevated levels of stress proteins	[77, 78]

Proteomic profiling of skeletal muscle biopsy material from patients suffering from type 2 diabetes	Establishment of a large number of muscle-associated signature molecules of type 2 diabetes; changed abundance in various mitochondrial proteins; abnormal phosphorylation of ATP synthase beta-subunit	[79–81]

Proteomic profiling of rectus abdominus tissue from obese and morbidly obese women with potential prediabetic side effects	Increased levels of key glycolytic enzymes suggests an obesity-related compensatory glycolytic shift in muscle metabolism	[87]

Proteomic analysis of gastrocnemius muscle from the nonobese Goto-Kakizaki rat model of type 2 diabetes	Changes in muscle proteins associated with the contractile apparatus, the antioxidant defense system, detoxification mechanisms, the cellular stress response, glucose metabolism, fatty acid utilization, nucleotide metabolism, and amino acid metabolism	[82, 83]

Subproteomic survey of the muscle mitochondria-enriched fraction from the non-obese Goto-Kakizaki rat model of type 2 diabetes	Differential expression of various mitochondrial marker proteins agrees with the idea that mitochondrial dysregulation plays a role in type 2 diabetes	[84]

Comparative proteomic study of fenofibrate-dependent protein expression in skeletal muscle from type 2 diabetic OLETF rats	Increased levels of the functionally unknown muscle protein C11orf59 in a fenofibrate-dependent manner in diabetic rat muscle	[85]

Proteomic analysis of obese and potentially prediabetic animal models	Generally perturbed protein expression levels in obese muscles, affecting especially metabolic enzymes	[88–92]
